# Teleproctoring of endovascular treatment of aortic diseases in times of pandemic and war

**DOI:** 10.1590/1677-5449.202400412

**Published:** 2025-02-21

**Authors:** Fabio Augusto Cypreste Oliveira, Fabio Lemos Campedelli, Carlos Eduardo de Souza Amorelli, Ana Luiza Pereira da Silva Pozzetti, Milena Fachini dos Santos Bossa, Priscila Cristina João

**Affiliations:** 1 Angiogyn, Goiânia, GO, Brasil.; 2 Universidade Estadual Paulista "Júlio de Mesquita Filho" – UNESP, São Paulo, SP, Brasil.; 3 Braile (International Sales Manager), São José do Rio Preto, SP, Brasil.; 4 Universidade Federal de São Paulo – UNIFESP, São Paulo, SP, Brasil.

**Keywords:** telemedicine, telementoring, teleproctoring, endovascular procedures, aorta, covid-19, war, telemedicina, telementoria, teleproctoria, procedimentos endovasculares, aorta, covid-19, guerra

## Abstract

Telemedicine encompasses various activities leveraging technology for remote health care. Surgical teleproctoring is described in the literature and has been used by some medical specialties for real-time and remote intraoperative guidance. However, there is still limited evidence on its use for the implantation of aortic endoprostheses. Technological progress and the advancement of telemedicine encourage surgical telementoring, especially in circumstances where in-person proctorship is not feasible, such as in remote areas, during pandemics and wars, and in settings lacking financial resources. In this context, we present the successful application of easily available and low-cost online teleproctoring of real-time aortic endoprosthesis implantation, addressing aortic diseases in a country experiencing social isolation due to the ongoing pandemic and war.

## Resumo

A telemedicina inclui múltiplas ações relacionadas ao uso da tecnologia para promoção de saúde de forma remota. A teleproctoria cirúrgica, já descrita na literatura, vem sendo utilizada por algumas especialidades médicas para orientação intraoperatória em tempo real e a distância. No entanto, ainda há poucas evidências para seu uso em implantes de endopróteses aórticas. O progresso tecnológico e o avanço da telemedicina fomentam a telementoria cirúrgica, especialmente em circunstâncias nas quais a proctoria médica presencial é inviável, tais como áreas remotas, pandemias, guerras e falta de recursos financeiros. Nesse contexto, apresentamos a aplicação bem-sucedida de teleproctoria em tempo real, facilmente acessível e de baixo custo, em implantes de endopróteses aórticas para o tratamento de doenças da aorta, em um contexto de isolamento social devido à pandemia e a uma guerra em curso.

## INTRODUCTION

Teleproctoring (TP) or surgical telementoring involves the exchange of guidance among surgeons through remote telecommunication technology.^1,2^ This method has been adopted by various surgical medical specialties to overcome challenges related to the availability of specialized medical teams and ensure effective and safe care for patients requiring specific procedures.^2-4^ TP enables surgeons unfamiliar with certain medical devices and recently developed surgical techniques to receive real-time intraoperative guidance, contributing to safe and effective surgeries and fostering development in surgical education and research.^3-7^

The COVID-19 pandemic and the imposition of social isolation accelerated advances in telemedicine, emerging as an essential tool for maintaining continuity of patient care and professional training.^8^ The ability to connect surgeons in different locations, even in challenging scenarios, demonstrates the versatility and growing importance of TP in modern surgical practice.^3,5^ This article reports the successful application of real-time TP to endovascular procedures conducted at distances exceeding 10,000 kilometers, connecting Brazil and Ukraine amid the COVID-19 pandemic and an ongoing war between Ukraine and Russia.

## METHODS

Endovascular procedures with TP were conducted following a protocol developed and routinely used by the specialized vascular surgery proctoring team Angiogyn (Goiânia – Goiás, Brazil), in collaboration with experts in endovascular devices, international relations, and compliance from Braile Biomédica® (São José do Rio Preto – São Paulo, Brazil). The study was approved by the local Ethics Committee (approval number 5.052.995).

The protocol for real-time TP of aortic endoprosthesis implantation included:

1. Receipt of the patient’s medical history and angiography images with 1mm slices.
2. Case analysis by the medical proctoring team, endoprosthesis specialists, and clinical engineering.
3. Definition of endovascular devices and surgical tactics to be used.
4. Teleconference among multidisciplinary teams with consensus on surgical planning (Figure 1) and online training in the use of endoprostheses and endovascular devices through institutional videos and clinical discussions between teams.

**Figure 1:**
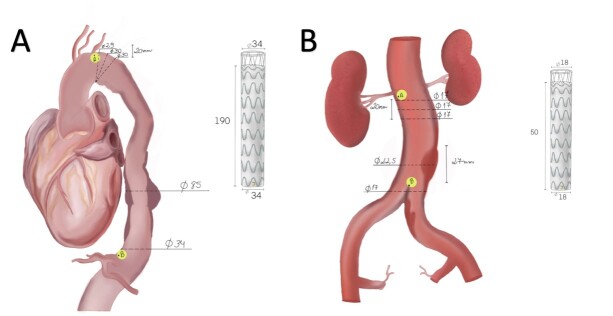
Surgical planning: schematic drawing of the endoprostheses to be used based on angiography analysis: A) Endovascular repair of the thoracic aorta (TEVAR) with a Braile Biomédica® DOMINUS® ENDOPROSTHESIS STENT-GRAFT and B) Covered Endovascular Reconstruction of the Aortic Bifurcation (CERAB) with a Braile Biomédica® custom abdominal straight endoprosthesis + 2 Advanta V12® balloon-expandable covered stents for both common iliac arteries.
5. Testing Internet connection and telecommunication devices in the hemodynamics sector the day before the procedure.
6. Retesting of connections and arrangement of telecommunication devices on the day of the surgical procedure (Figure 2).

**Figure 2:**
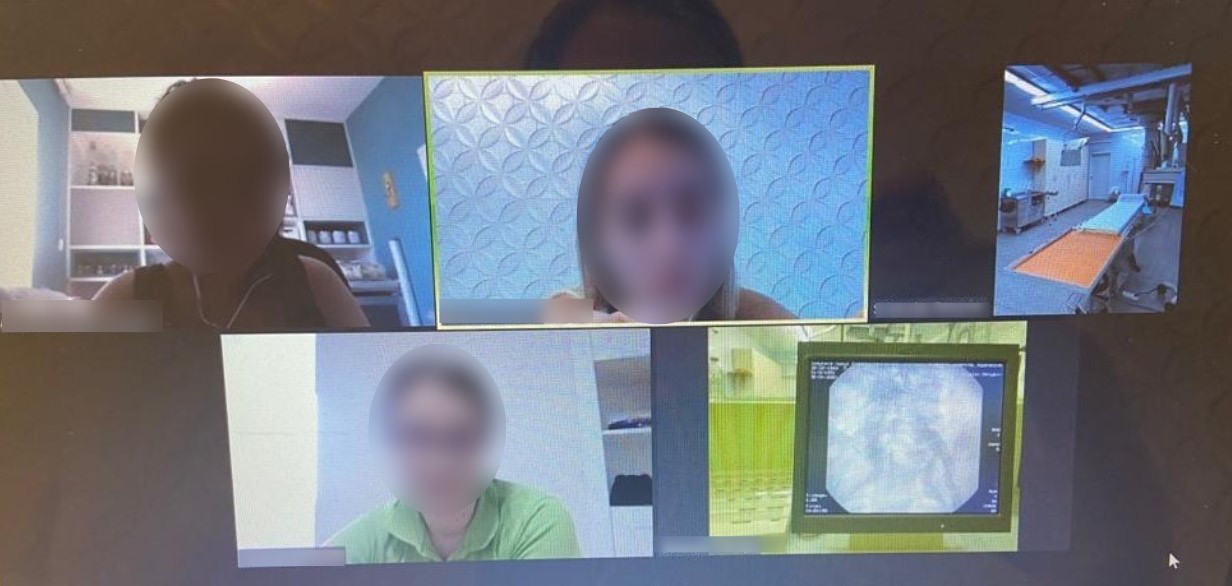
Testing connections and arrangement of telecommunication devices on the day of the surgical procedure.
7. Execution of the surgical procedure.

**Figure 3:**
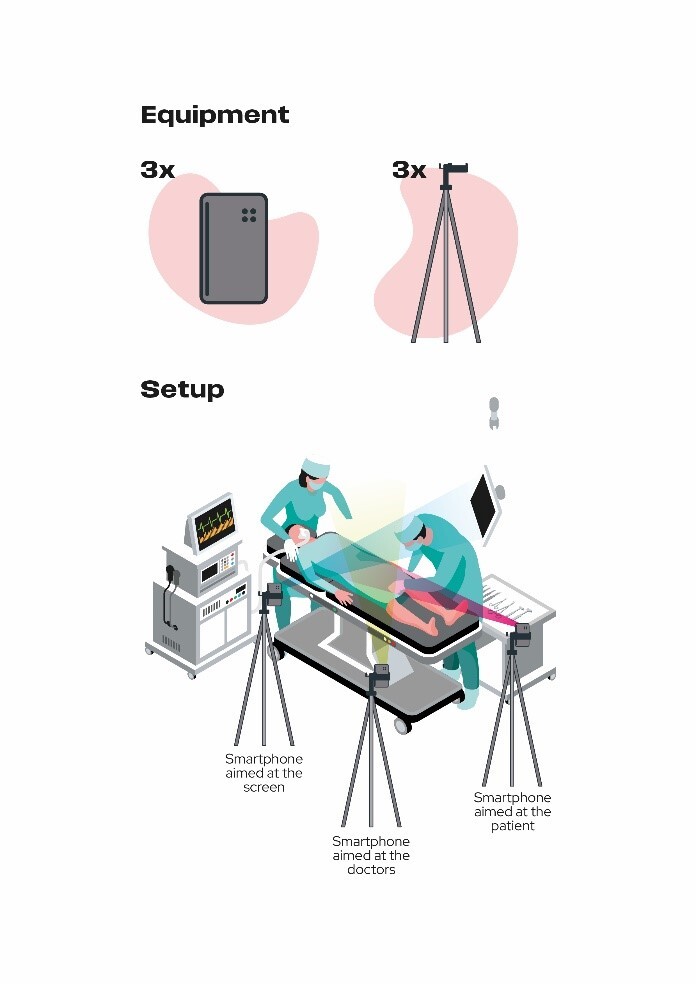
Illustration of teleproctoring setup employed.

**Figure 4:**
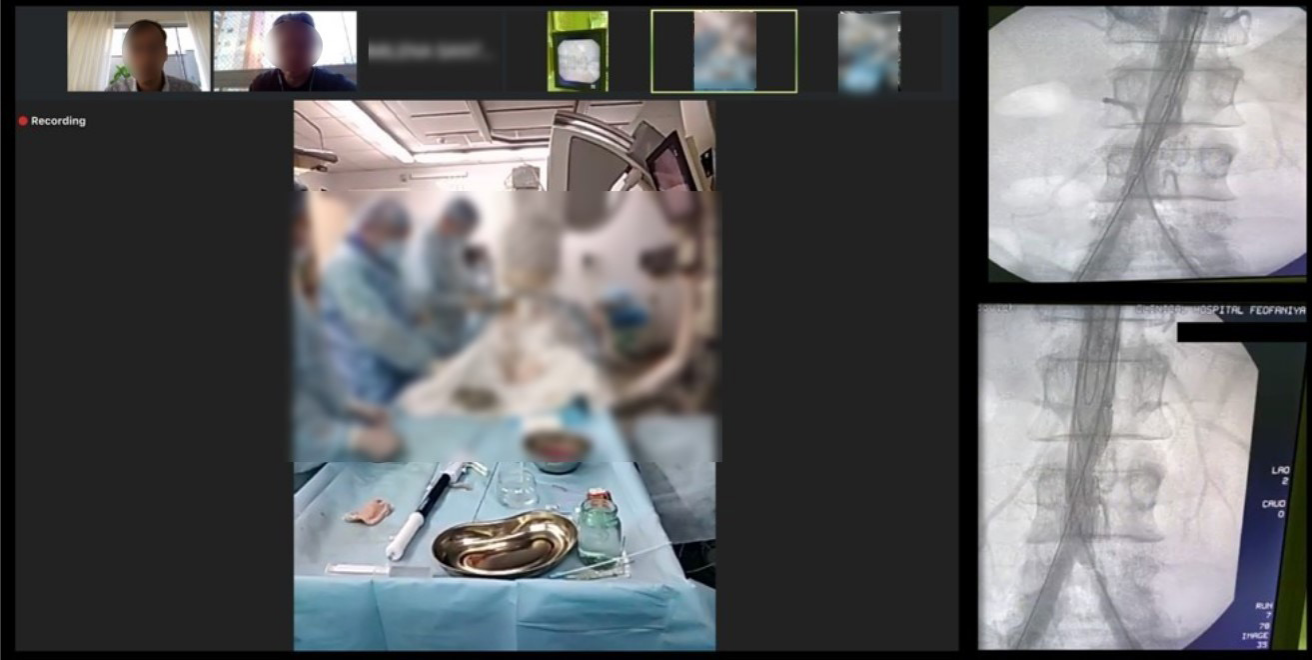
Implantation of the Braile Biomédica® DOMINUS® ENDOPROSTHESIS STENT-GRAFT with TP assistance to treat a thoracic aortic aneurysm.

**Figure 5:**
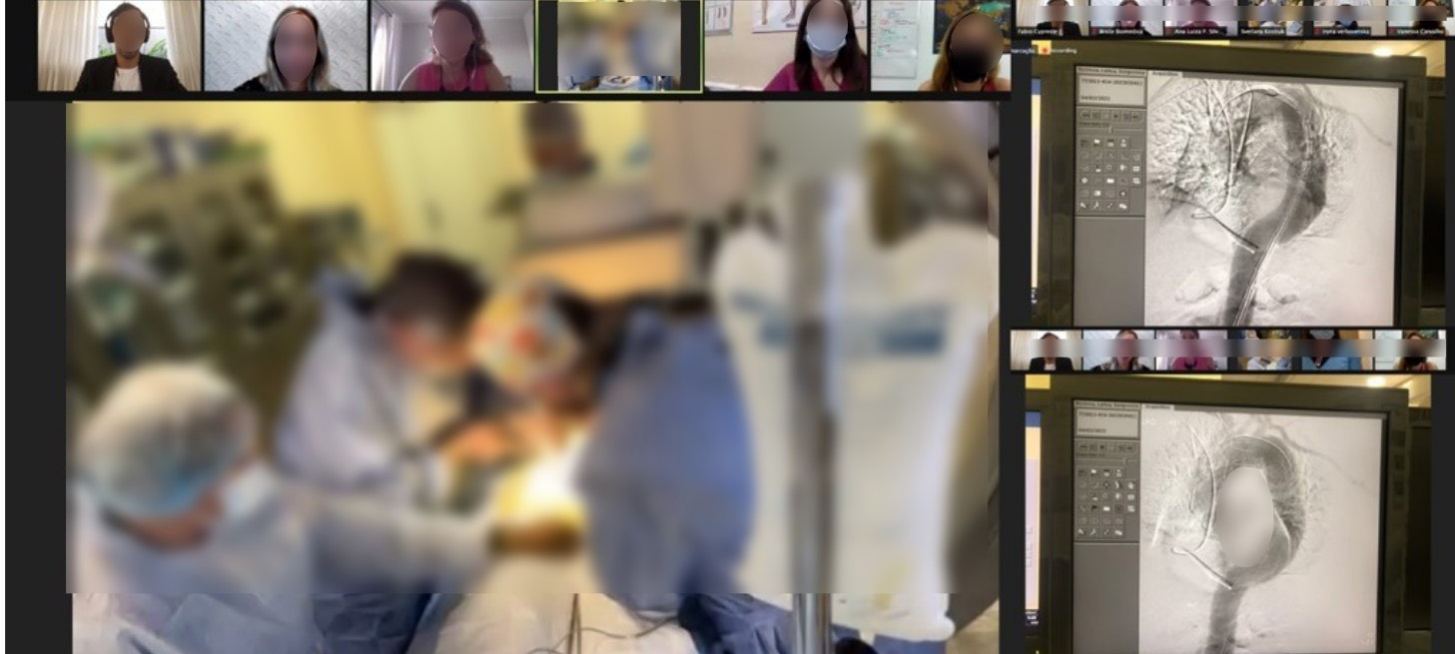
Implantation of a Braile Biomédica® custom abdominal endoprosthesis to treat a penetrating abdominal aortic ulcer.

Following the described protocol, endovascular procedures were conducted using real-time TP through an online platform. Bidirectional and encrypted communication between the multidisciplinary team in Brazil and Ukraine was established over a shared broadband 4G internet service, with high-resolution imaging (1280x720). Three telecommunication devices were strategically positioned with tripods, providing the Brazilian team with a comprehensive view of the fluoroscopy monitor, the Ukrainian team, and the handling of endoprostheses in both cases (Figure 3). The TP team consisted of three members (a proctor and two product specialists), while the local team performing the procedure included two cardiovascular surgeons and an assistant who facilitated communication with the Brazilian team. The assisting surgeon maintained direct bidirectional communication with the Brazilian teleproctoring team through a headset. The first TP-assisted case involved a 62-year-old, white, hypertensive female who was a smoker. She presented with a penetrating aortic ulcer accompanied by an intramural hematoma and a thoracic aortic aneurysm in Zone 4. The procedure involved implantation of a DOMINUS® stent-graft endoprosthesis (Figure 4). The second case involved a 56-year-old, white, hypertensive, diabetic male, who was also a smoker. He was diagnosed with aortic stenosis and a penetrating abdominal aortic ulcer. The procedure involved implantation of a custom-made abdominal endoprosthesis (Figure 5).

## RESULTS

TP achieved efficacy and safety for conducting endovascular procedures. Technical success, defined as the correct deployment of planned devices without the need for repositioning and/or the use of unplanned devices, was achieved in both cases. Additionally, clinical success, defined as absence of death and clinical complications related to endovascular procedures conducted with TP within 30 days, was also achieved. The duration of the TP real-time connection was 34 minutes for the TEVAR procedure and 1 hour and 4 minutes for the CERAB procedure. Both procedures were conducted under general anesthesia with femoral access via surgical dissection, using approximately 100 ml of iodinated contrast. No adverse events occurred during either of the procedures. Both the field team and the proctoring team expressed high satisfaction with the outcomes and are confident in the potential for replication of this approach in other cases.

**Figure 6:**
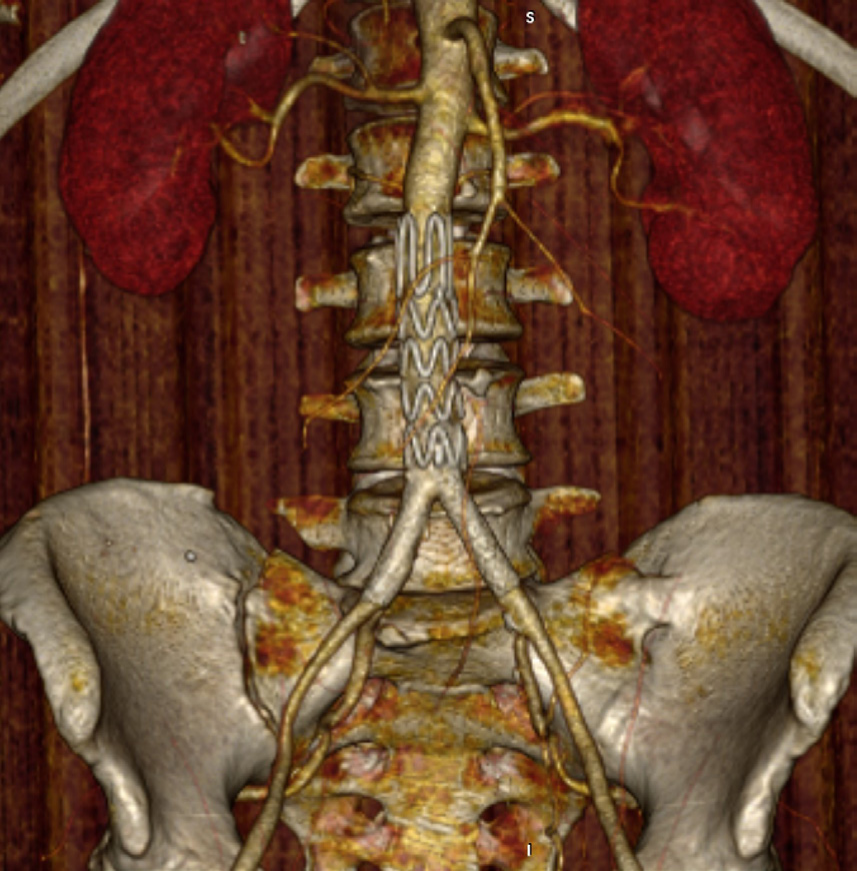
3D reconstructed angiography demonstrating correct positioning of the endoprostheses and complete exclusion of the penetrating abdominal aortic ulcer with no leakage.

There were no observed difficulties with video resolution or latency between the teams in either of the cases. Postoperative control angiography at 30 days showed complete resolution of the aortic lesions that motivated treatment, with no signs of leakage, thrombosis, or migration of the endoprostheses used in either case (Figure 6).

## DISCUSSION

After the COVID-19 pandemic, significant changes and advances in education and care were observed in the field of surgical medicine. These include the growth of teleconferences, webinars, distance education, and the use of simulations and virtual reality in medical training. Advances in surgical teleproctoring, driven by telemedicine, have facilitated training of new specialists, especially in regions with a shortage of senior surgeons, providing access to procedures and surgical training in remote areas, contributing to the improvement of advanced surgical techniques, and consequently achieving better results for both the medical team and the patients. While there are various studies of the use of telemedicine in vascular surgery, specific literature on TP of vascular surgeries is still limited.^5,6,7,9^

The first report in the literature that employed TP to assist in aortic endoprosthesis implantation was published by Deaton et al. in 1999.^6^ Using remote transmission links, the team supervised and monitored the proximal and distal release and final control angiography in the endovascular treatment of abdominal aortic aneurysms (EVAR) in 7 patients.^6^ Since then, several studies have explored this approach, confirming its feasibility and safety.

In 2005, Di Valentino et al.^7^ conducted a prospective study to assess the safety of TP during EVAR. The study compared three proctoring modalities: in-person monitoring for 12 patients, TP in a room adjacent to the operating room for another 12 patients, and remote TP (250 km away) for a group of 24 patients. Surprisingly, no significant differences were identified between the groups regarding proctoring time or procedure success. The authors concluded that TP for EVAR is not only feasible but also shows promising results. They encouraged the replication of this surgical proctoring model for other invasive cardiovascular procedures.

Another study, in 2017, investigated the safety of implementing a telementoring program for EVAR.^9^ During the initial training phase, 49 patients underwent EVAR at the secondary center under telementoring. Subsequently, the same center independently performed 86 EVAR cases. The results revealed no significant difference between telementored and independent procedures, either in terms of 30-day mortality (4.1% vs. 2.3%, p=0.621) or in terms of initial technical success (93.9% vs. 97.7%, p=0.353). The study concluded that telementoring for EVAR in a remote center is effective and feasible, contributing to the successful implementation of medical competence in a distant environment.

The essential conditions for safe TP were outlined by the Society of American Gastrointestinal and Endoscopic Surgeons (SAGES) at an event called Project 6 Summit.^10^ The criteria established for telecommunication systems for telementoring included rigorous requirements such as low latency, stability, minimum video resolution of 480 scan lines, and a minimum broadband connection of 512 kbps, along with the essential encryption of all data to ensure compliance with the Health Insurance Portability and Accountability Act (HIPAA) security standard. The committee also emphasized desirable qualities for telementoring, highlighting the need to be low-cost, "plug and play," easy to use, portable, upgradable, and enabling telestration, which is the ability to draw and annotate on videos and images.

In 2021, Gerardo et al.^4^ further advanced the specifications, stating that for surgical telementoring, image resolution should not be less than 768x492, and video transmission latency should not exceed 25ms. This implies that a broadband connection greater than 1.2mbps is imperative to ensure the effectiveness of surgical telementoring. These guidelines reiterate the importance of specific technical conditions to ensure the quality and safety of TP.

In the presented cases, TP of endovascular treatment of aortic diseases with endoprosthesis implantation was used by experienced surgical teams unfamiliar with the specific devices used. Minimum criteria for data transmission, security, and resolution were met, even using easily accessible devices not specifically dedicated to TP. Additionally, telestration was not necessary in either case, despite being available.

## CONCLUSION

Remote real-time teleproctoring, using readily available and low-cost technology, proved effective for endoprosthesis implantation to treat complex aortic diseases in a country undergoing healthcare and social isolation. The role of remote surgical teleproctoring in endovascular procedures is still evolving. However, it seems to be an irreversible path for improving global healthcare assistance and surgical training, leading to reductions in time, distance, and costs.
